# Back to the future: lessons from past viral infections and the link with Parkinson’s disease

**DOI:** 10.1042/NS20200051

**Published:** 2021-04-16

**Authors:** Eilis Dowd, Declan P. McKernan

**Affiliations:** Pharmacology and Therapeutics, School of Medicine, National University of Ireland, Galway H91 TK33, Ireland

**Keywords:** Neurodegeneration, Neuroinflammation, Parkinson's disease, SARS-CoV2, Viral infection

## Abstract

During the current coronavirus disease 2019 (COVID-19) pandemic, there has been noticeable increase in the reporting of neurological symptoms in patients. There is still uncertainty around the significance and long-term consequence of these symptoms. There are also many outstanding questions on whether the causative virus, severe acute respiratory syndrome coronavirus 2 (SARS-CoV2) can directly infect the central nervous system (CNS). Given the long association between viral infections with neurodegenerative conditions such as Parkinson’s disease (PD), it seems timely to review this literature again in the context of the COVID-19 pandemic and to glean some useful information from studies on similar viruses. In this commentary, we will consider the current knowledge on viral infections in the brain. In addition, we review the link between viral infection and neurodegeneration in PD, and review the recent literature on SARS infections, the potential link with PD and the potential areas of study in the future.

In 1918–1920, Influenza A H1N1 caused devastation to the world’s population killing an estimated 50 million people [1]. In addition, many who survived this pandemic developed long-term health complications. A link between viral infection and Parkinson’s disease (PD) was described in the early 1930s [2]. It was subsequently reported that many people who survived the 1918 H1N1 Influenza A epidemic developed a type of sleeping sickness which also included nausea, headache, fever, catatonia and even coma. This sickness was known as encephalitis lethargica (EL) [3]. In the midst of the current coronavirus disease 2019 (COVID-19) pandemic, (where there were over 60 million cases and 1.4 million deaths worldwide at the time of writing) it is important to ask if similar events will take place in the future and to prepare ourselves for the long-term healthcare implications [4,5]. We will briefly review the current knowledge on central nervous system (CNS) infections by viruses, the link between PD and viral infections and finally what lessons can be learned going in the future once the current pandemic is over.

## Viral infection of the CNS

At present, we know comparatively little about viral infections of the CNS compared with other systems and organs due to the accessibility of brain. We have some examples of viruses with strong neurotropisms and these have been detected in brain parenchyma *post-mortem* while other viruses that seem to have no obvious neurotropisms but that cause neurological symptoms. These can be dsDNA and ssRNA viruses that are segmented or enveloped with a variety of incubation periods. They come from a variety of different families including *Togaviridae, Bornaviridae, Bunyaviridae, Flaviviridae, Herpesviridae, Orthomyxoviridae, Paramyxoviridae, Picornaviridae* and *Rhabdoviridae.* Some of these viruses, such as those from *Herpesviridae* family, can establish latency and cause illness following reactivation [6]. These can suppress the cytokine and interferon responses by inhibiting the activation of transcription factors or receptors involved in signalling pathways [7] .

In addition to those viruses with strong neurotropism, there are also a number of respiratory and enteroviruses that can eventually infect the brain such as influenza and cocksackie B virus respectively [6,8–10]. CNS diseases are the most common extra-respiratory complication with flu. These can include meningitis, encephalitis, encephalopathies and febrile seizures [11]. A review of the influenza epidemics and pandemics of various different strains in the past 100 years has shown many of the various neurological symptoms reported post infection including encephalitis, encephalopathy, seizures and delirium [12].

Neurotropic viruses have been reported to spread following infection of axon termini of sensory and autonomic neurons from various mucosal epithelia to the spinal cord via retrograde axonal transport. Other viruses may also use retrograde transport to make their way from the neuromuscular junctions of motor neurons at skeletal muscle via the spinal cord and eventually to the brain. A second option for neuronal transport is via the olfactory nerve. This can often be used by viruses present in the airway mucosa directly infecting the olfactory neurons in the upper respiratory tract. From there anterograde axonal transport may spread the virus through the cribriform plate and into the olfactory bulb and from there into other brain regions. A potential non-neuronal route for neurotropic viruses may be via the blood–brain barrier. Immune cells infected with virus may attach to the brain endothelial cells and traverse across this layer and then come into contact with neurons. This may also occur with virus at the meningeal–cerebrospinal barrier and with virus present within blood vessels at the choroid plexus [13–15]. Infection with some neurotropic viruses has been reported to lead to encephalitis which can be fatal in some cases [6]. Given the experience of the past, very recent studies have now focussed on the tracking the potential long-term effects of CNS infection and the potential that these infections predispose individuals to neurodegenerative and neuropsychiatric conditions.

## Viral infection and PD

PD is the second most common neurodegenerative disorder and most common neurodegenerative motor disorder. It is associated with degeneration and eventual loss of dopaminergic neurons from the substantia nigra pars compacta of the midbrain. Mitochondrial dysfunction, oxidative stress, microglia and astrocyte activation leading to, autophagy disruption and uncontrolled protein aggregation involving α-synuclein have also been described [16,17]. Neuroinflammation has been implicated in the progression of this disease, by which microglia become chronically activated in response to α-synuclein pathology and dying neurons, thereby acquiring phenotypes that are cytotoxic and can cause further neuronal death [18,19]. This results in a significant reduction in dopaminergic activity in the nigrostriatal pathway contributing to disease symptoms such as resting tremor, rigidity, bradykinesia and unstable posture. Non-motor symptoms such as olfactory dysfunction, dysphagia, constipation, sleep disturbances and dementia have also been described [20].

While genetically inherited mutations can explain 5–10% of PD cases, the root cause(s) of nearly 90% of cases so-called idiopathic PD remains unknown. Infection has been suggested as a potential cause although perhaps not the only. Other suggestions include occupational pesticide or heavy metal exposure and traumatic brain injuries. These in addition to predisposing genetic polymorphisms may accelerate the development of this disease [16,17]. Having reviewed the literature on different infections and PD recently [8], we will now review the literature on the potential role of viral infection in PD, with more of a specific focus on data from the recent epidemic involving the severe acute respiratory syndrome coronavirus 2 (SARS-CoV2) that leads to COVID-19.

A recent meta-analysis reported that infectious disease (including bacterial and viral infections) increased the risk of PD by 20% [21]. Another meta-analysis investigating infection-related risk of PD with 13 different bacterial, fungal and viral organisms found that those infected with *Helicobacter pylori*, hepatitis C virus (HCV), *Malassezia* and *Chlamydophila pneumonia* had an increased risk of developing PD while those with infections caused by influenza, herpes, hepatitis B (HBV), scarlet fever, mumps, measles and German measles had not. It was also found that antiviral therapy against HCV reduced the risk of PD in those patients [22].

There have been a number of studies reporting Parkinson’s-like symptoms years after infection with individual viruses including Herpes simplex virus (HSV), influenza A, measles, cytomegalovirus and mumps [23]. Many viral infections that cause encephalitis, have been linked with a post-encephalitic parkinsonism (PEP) including human immunodeficiency virus (HIV), coxsackie virus and Japanese encephalitis B virus [24]. It is important to state at this stage that these infections did not lead to classical PD but Parkinsonism. Those diagnosed with PEP typically do not display Lewy body deposition on examination *post-mortem* [25].

Human studies have their limitations with regard to delineating a pathophysiological mechanism. On the other hand, animal models have allowed some further insight into the potential mechanisms involved. It has been reported that when mice were infected with H5N1 influenza virus, it progressed from the peripheral nervous system into the CNS where it was able to activate the innate immune system in the brain and this led to long-term activation of microglia and some dopaminergic cell loss [26]. Neuroinflammation and microglial activation was also seen in an intranasal infection model used with the H1N1 strain of influenza [27]. It also was demonstrated to cause α-synuclein aggregation [28]. When H1N1 was combined with the known Parkinsonian toxin MPTP (1-methyl-4-phenyl-1,2,3,6-tetrahydropyridine) there was also a cumulative loss of dopaminergic neurons in substantia nigra pars compacta greater than each agent on their own [29].

It has been suggested from clinical studies that viruses like HSV lead to disease progression by a form of molecular mimicry by inducing what seems like an autoimmune response by the body. HSV is known to remain latent in the brain for many years but once reactivated may induce antibodies that can cross-react with α-synuclein sequences, which may also induce aggregation of the peptide and also potentially induce a T-cell response in such cases [30,31]. Antibody cross-reactivity and α-synuclein aggregation has also been reported with Epstein–Barr virus infection [32] and loss of neurons in the basal ganglia has also been reported in this infection [33].

A recent meta-analysis reported an increased risk of Parkinson’s in those infected with HCV [34]. CNS infection may be facilitated by the expression of HCV receptors on brain microvascular endothelial cells of the brain [35]. HCV infection was shown to induce neuroinflammation and neuronal death in animal models [36]. Japanese encephalitis virus infection of rats causes decreases in striatal dopamine levels as well as significant loss of tyrosine hydroxylase (TH) positive neurons [37]. While many cell types within the brain can be infected by various viruses, there may be some selectivity for certain viruses. For example, for infection with West Nile virus (WNV) this selectivity may depend on the expression of α-synuclein which also increases on infection. It was reported that α-synuclein could localise with WNV envelope antigen, influence ER signalling and could inhibit neuronal infection [38]. This could have long-term consequences for those infected and increase the possibility of developing PD in the long-term if it does indeed increase expression of α-synuclein in the long term [39].

We know from both inflammatory animal models of PD and from clinical studies that neuroinflammation is an important component of disease progression in PD [40–42]. It has also been documented that there is an increase in peripheral inflammation in PD patients. This low-level chronic inflammation may be a driver for inflammation at the blood–brain barrier causing disruption and entry of pathogens [43–45]. Meta-analyses of peripheral cytokine levels indicated that levels of interleukin (IL) 6 (IL6), IL1β, tumour necrosis factor (TNFα), IL10, IL2, regulated upon activation, normal T cell expressed and presumably secreted (RANTES) and C-reactive protein (CRP) are significantly elevated in PD patients compared with healthy controls [46,47]. Other analyses suggest these elevations are predictive of disease progression particularly for motor and cognitive symptoms [48]. Looking beyond the current pandemic, it will be important to fully investigate if these same events occur with SARS-CoV2 infection.

## Coronaviruses and PD

SARS-CoV2 infection leads to the disease known as COVID-19. SARS-CoV2 is an enveloped β coronavirus that shares a significant amount of genetic similarity with other family members such as SARS-CoV (approx. 80%). Its viral envelope contains a spike glycoprotein (S) which it uses to gain entry to human cells via the membrane-bound angiotensin converting enzyme 2 (ACE2). It also seems to require proteolytic processing by transmembrane protease serine 2 (TMPSS2) for cell entry. It has proofreading capacity unlike some other RNA viruses and therefore thought to have lower mutation rates. It also has higher infectivity than SARS-CoV due to structural differences in its surface proteins that means it has greater affinity for ACE2 [49]. Hyperinflammation is a key component of COVID-19 with significant increases in the plasma concentrations of CRP, IL-6, IL-8, IL-10, IL-2R and ferritin [50] as well as altered ratios of monocyte subsets and B and T lymphocytes at different disease severities [51].

At present, there is not enough concrete evidence proving that SARS-CoV2 infection is a causative factor in PD or Parkinsonism. In the past number of months, case reports and some small studies have linked SARS-CoV2 infection to a variety of neurological symptoms including stroke, encephalopathies, encephalitis, meningitis, confusion, cognitive deficits all with varying severities [52–56]. It will be important to further explore the mechanisms behind these symptoms. CNS infection by the *Coronaviridae* family has been known about for some time, with several studies detailing the pathogenic strategy, kinetics and host immune responses for members of this family (reviewed in [57]). These viral infections have been associated with stroke, seizure, convulsions and encephalitis [58,59]. During infection with some family members including mouse hepatitis virus, resolution of infection results in a chronic CNS disease with low-grade inflammation. During this period, infectious disease remains undetectable but viral antigens persist [60]. Other members of the coronavirus family have already been linked with PD. Antibodies to mouse as well as the human coronaviruses OC43 and 229E (those that cause the common cold) were found in the CSF of some although not all patients with PD were tested [61]. It was reported that OC43 virus may travel between neurons by axonal transport in a mouse model of infection [62]. There were also several case reports linking Middle East respiratory syndrome coronavirus (MERS-CoV) and neurological symptoms in patients [63,64], human coronavirus (HCoV) with acute disseminated encephalomyelitis (ADEM) in a child [65] as well as fatal encephalitis with coronavirus OC43 in a child [66].

**Figure 1:**
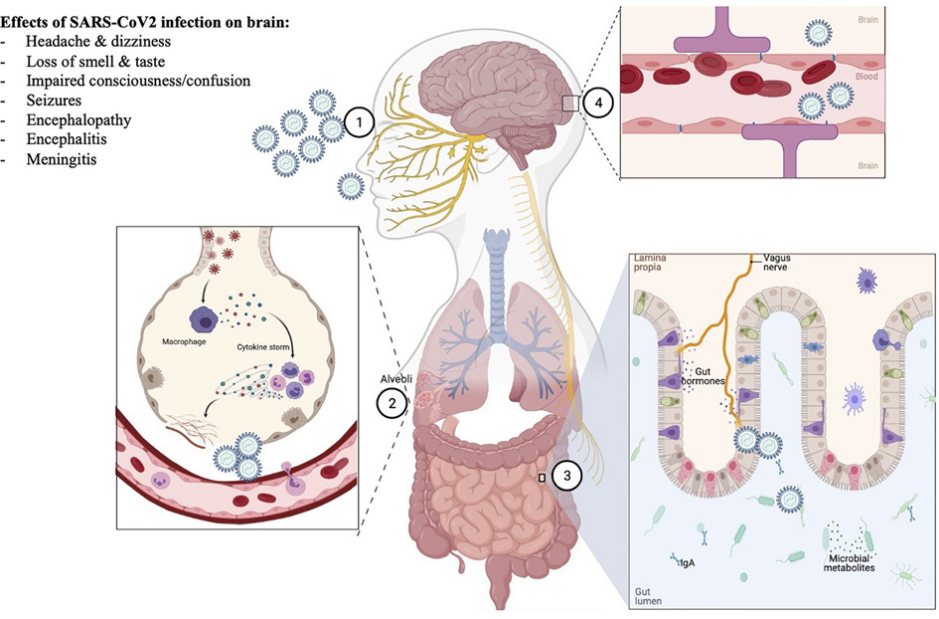
Potential routes of entry of SARS-CoV2 and effects on brain function Shown in this figure are the potential routes of entry of SARS-CoV2 to the brain numbered 1–4. Route 1 is via the nasal epithelium following inhalation and transport via olfactory nerve, route 2 is via the alveoli following inhalation into the lungs, route 3 is via the gastrointestinal tract following ingestion and transport via the vagus nerve, route 4 is via the blood–brain barrier. Highlighted in box 1 are the most common conditions reported to date following SARS-CoV2 infection.

While there are obvious links between SARS-CoV2 and organs like the lungs, the link with the CNS is less obvious (see Figure 1). There has been a recent increase in the number of studies examining the potential tropism of this virus for the CNS. In particular, attention has focussed not only on the presence of viral RNA and protein but also the expression of receptors that will facilitate viral invasion such as ACE2 and TMPRSS2 [15]. ACE2 receptors are highly expressed in epithelial cells lining the respiratory and mucosal tracts, their expression can be regulated by interferon signalling and they have an important role in the renin–angiotensin system [67]. *Ex vivo* studies human brain organoids suggest that blockade of ACE2 receptors with antibodies from the CSF of patients can prevent neural infection [68]. With previous coronaviruses such as SARS-CoV there have been reports of viral particles in the brain in addition to other organs [69,70]. For SARS-CoV2, recent *post-mortem* studies detected viral RNA and protein in the brain although the regions were not specified and in many studies only in a subset of patients [68,71,72]. The routes of invasion are still uncertain but there has been some evidence of blood–brain barrier disruption from the analysis of CSF from infected patients [73]. There has been a suggestion that given the sequence similarity to SARS-CoV that invasion of the cardiorespiratory centres of the medulla may contribute to the respiratory failure seen in those severe infection [74]. There has been some evidence recently from a *post-mortem* case series which found evidence of microglial activation and T-cell infiltration of the brainstem, cerebellum and meninges [75]. While other studies suggest an involvement of astrocytes and disruption of brain metabolism [76]. Further studies will need to be carried out to add this evidence.

Neurological symptoms that have been reported to date in patients with COVID-19 include stroke, seizure, headache, fatigue, confusion, insomnia, anosmia, ageusia, neuropathic pain and myalgia among others [77]. In particular, anosmia and ageusia are thought to be very common and are included as in the list of major symptoms [78]. Coincidentally, both these symptoms are classical prodromal features of PD [20]. There have been initial case reports that purport a link between the virus and PD, describing decreased dopamine uptake in the putamen [79–81]. This in itself does not prove the virus as the causative agent. These are single case reports and these events may have coincided with the commencement of the prodromal phase of the condition in these individuals. There have since been case reports showing the presence of viral particles in the brain and capillary endothelium of COVID-19 patients *post-mortem* [82]. SARS-CoV2 infection was reported to lead to elevated plasma levels of neurofilament light chain protein (NfL) and glial fibrillary acidic protein (GFAP) two markers usually indicative of CNS injury in patients with COVID-19 [83].

## Future perspectives

Given that the SARS-CoV2 virus was only identified and genetically sequenced in the past year [84,85], there has been a significant amount of progress made in characterising and understanding the viral pathogenesis and in developing therapies and vaccines. However, there are still many questions left unanswered at present. One of these is whether the virus is present or not in the brain parenchyma. Following on from this, if it is present is it a selective process and does it vary from patient to patient? If it is present, how does it gain access and are there particular tropisms for neurons or glia or both? Is entry correlated to expression levels of ACE2/TMPSS2 or are there alternative receptors by which it can gain entry? Is entry via a neuronal or haematogenous route? If it is not present in significant amounts, how does it lead to such pathologies in patients? Is there a role for the immune response in such cases? It is now imperative to design future studies to discover these answers.

Many of the case reports and small studies highlighted here were conducted on *post-mortem* tissue in those who had the illness for a short period. What is needed now is long-term follow-up neurological studies on those who survived COVID-19. It will also be of interest to compare those who may have been asymptomatic, who had mild illness, moderate illness and those who were hospitalised or on mechanical ventilation to determine if the severity of symptoms or even the viral load has an effect on the severity and type of neurological injury. Imaging techniques could also potentially be developed to identify the presence of binding sites for the virus and their locations. It is already mentioned that antibodies to other viruses are present in PD patients [61]. We are also uncertain as to the long-term consequences of infection on motor and non-motor symptoms relevant to PD. Will previous infection predispose individuals to these symptoms sooner or will there be greater severity of symptoms? Long-term follow-up studies will be needed. It will be important to design studies on the measurement of antibodies to SARS-CoV2 in such cohorts. It will also be important to determine if such antibodies play a role in the pathophysiology of PD via molecular mimicry as has been suggested with studies of other viral infections such as Epstein–Barr [32].

The use of validated animal models should also play their part. At present, there is not enough understanding about what regulates the expression of binding proteins such as ACE2 in different tissues and if expression levels increase the risk of brain infection. There is also a need to determine the potential number of routes of infection as well as the role of the immune system in the periphery as well as the CNS in neurological symptoms. These could lead to the development of targeted therapies for the brain. It was shown previously that antiviral drugs and vaccination may potentially alleviate the symptoms in an animal model of influenza infection [29]. Could this also have consequences for the SARS-CoV infection? Many questions remain and the next 5–10 years will require a considerable investment not only in vaccines but also in the basic and clinical research studies that will be needed to track the long-term complications of this illness.

## Abbreviations

ACE2: angiotensin converting enzyme 2
CNS: central nervous system
COVID-19: coronavirus disease 2019
CRP: C-reactive protein
HCV: hepatitis C virus
HSV: Herpes simplex virus
IL: interleukin
PD: Parkinson’s disease
PEP: post-encephalitic parkinsonism
SARS-CoV2: severe acute respiratory syndrome coronavirus 2
TMPSS2: transmembrane protease serine 2
WNV: West Nile virus
